# The Effect of Latent Tuberculosis Infection on Ovarian Reserve and Pregnancy Outcomes among Infertile Women Undergoing Intrauterine Insemination: A Retrospective Cohort Study with Propensity Score Matching

**DOI:** 10.3390/jcm12196398

**Published:** 2023-10-07

**Authors:** Yifan Chu, Ying Chen, Wen Yao, Luyao Wang, Bo Zhang, Lei Jin, Jing Yue

**Affiliations:** 1 Department of Reproductive Medicine, Tongji Hospital, Tongji Medical College, Huazhong University of Science and Technology, Wuhan 430030, China; chuyifan@hust.edu.cn (Y.C.); d202081960@hust.edu.cn (W.Y.); m202276476@hust.edu.cn (L.W.); bo.zhang@tjh.tjmu.edu.cn (B.Z.); ljin@tjh.tjmu.edu.cn (L.J.); 2 Department of Obstetrics, Tongji Hospital, Tongji Medical College, Huazhong University of Science and Technology, Wuhan 430030, China; d202282194@hust.edu.cn

**Keywords:** latent tuberculosis infection, ovarian reserve, pregnancy outcomes, intrauterine insemination, propensity score matching

## Abstract

Latent tuberculosis infection (LTBI) widely exists in patients with unexplained infertility, and whether LTBI would affect the ovarian reserve and pregnancy outcome of infertile women undergoing intrauterine insemination (IUI) is still unknown. A single-center, retrospective, cohort study was designed that included infertile women undergoing IUI at the Department of Reproductive Medicine, Tongji Hospital, Tongji Medical College, Huazhong University of Science and Technology in Wuhan, China, from January 2018 to December 2020. The primary outcomes of this study were ovarian reserve and live birth rate. Secondary outcomes included pregnancy outcomes and maternal and neonatal complications. As a result, 3066 IUI cycles were eventually enrolled in this study. Of these women, 9.6% (295/3066) had LTBI evidence. After propensity score matching (PSM), there was no significant difference in the baseline between the LTBI and non-LTBI groups. The data showed that women who had LTBI had trends toward lower biochemical pregnancy rates (12.9% vs. 17.7%, *p*-value 0.068), lower clinical pregnancy rates (10.8% vs. 15.1%, *p*-value 0.082) and lower live birth rates (8.1% vs. 12.1%, *p*-value 0.076), with no significant differences. There were also no significant differences in ovarian reserve and other secondary outcomes between the two groups. In conclusion, there were no significant differences in ovarian reserve, perinatal or neonatal complications between women with and without LTBI. Women with LTBI tended to have worse pregnancy outcomes after receiving IUI, but the difference was not significant.

## 1. Introduction

Tuberculosis (TB), caused by bacillus Mycobacterium tuberculosis, is a communicable disease that is a major cause of ill health and one of the leading causes of death worldwide. In 2019, TB was the leading cause of death from a single infectious agent, ranking above HIV/AIDS. The World Health Organization (WHO) reported that about 10.6 million people fell ill with TB and 6.4 million people are newly diagnosed with TB in 2021, and there were an estimated 1.6 million deaths in 2021 due to TB. The TB incidence rate (new cases per 100,000 population per year) rose by 3.6% between 2020 and 2021 [1]. Meanwhile, the novel coronavirus (COVID-19) pandemic continues to have a damaging impact on TB diagnosis and treatment and the burden of TB disease worldwide. China has made substantial progress in diagnosing and treating TB [2]. Nevertheless, it still has the third largest number of cases worldwide, with a TB incidence rate of 55 per 100,000 people in 2021 [1].

Latent TB infection (LTBI) is defined by a positive test for infection and no active TB evidence [3,4]. A recent study showed that the prevalence of LTBI in China was 18.8% [5]. The incidence of LTBI varies among different populations. LTBI prevalence among household contacts varied from 32 to 48% [6,7]. The TB infection rate in healthcare workers was found to range from 15% to 70% [8,9]. IGRA positivity was observed to be lower than 10% in schoolchildren and adolescents [10,11]. LTBI is insidious and atypical, and most women will even be infected without experiencing any symptoms. Some women may be infertile due to damage to the fallopian tubes, endometrium and ovaries caused by latent genital tuberculosis infection (LGTBI) [12,13,14]. Therefore, early diagnosis of TB is essential.

With the increasing incidence of infertility worldwide, assisted reproductive technology (ART) is widely used around the world. Intrauterine insemination (IUI) is the first and primary assisted reproductive treatment for couples with unexplained or mild male-factor infertility because it is thought to improve pregnancy opportunities moderately while being simple, easy to manage and relatively low cost [15,16]. Whether LTBI would affect the ovarian reserve and pregnancy outcome of infertile women undergoing IUI is still unknown. Therefore, we performed this retrospective analysis to evaluate the effect of LTBI on ovarian reserve and pregnancy outcomes.

## 2. Materials and Methods

### 2.1. Study Design

This was a retrospective cohort study of women who had undergone IUI for infertility from January 2018 to December 2020 at the Department of Reproductive Medicine, Tongji Hospital, Tongji Medical College, Huazhong University of Science and Technology in Wuhan, China. A total of 3295 IUI cycles (2459 artificial inseminations by husband (AIH) cycles and 836 artificial inseminations by donor (AID) cycles) were screened. All of the women had received an interferon-gamma release assay (IGRA) examination before IUI for TB screening. If the IGRA examination result is positive, C-reactive protein, erythrocyte sedimentation rate and chest X-ray or CT would be applied to determine whether women suffered from active TB. Furthermore, every woman in our department had undergone hysterosalpingography (HSG) or hysterosalpingo-contrast sonography (HyCoSy) examination before IUI to confirm the patency of at least one fallopian tube. If both fallopian tubes are obstructed and/or absent, the patient would be recommended for in vitro fertilization and embryo transfer (IVF-ET) treatment. Detailed information was obtained from the case registration research database. Exclusion criteria were: (i) active TB; (ii) cancellation; (iii) poor semen quality after optimization; (iv) critical data missing; and (v) switch to IVF-ET. A special team was assigned to follow the pregnancy outcomes. Clinical data were collected from the start of the IUI cycle to 6 weeks after delivery.

### 2.2. Etiologies of Infertility

According to the WHO and the Chinese manual for the standardized investigation and diagnosis of infertile couples, the etiologies of infertility were classified as male factors, female fallopian tube and pelvic factors, ovarian factors, endometriosis, uterine factors, chromosome factors and unexplained or other factors [17,18,19]. Since polycystic ovary syndrome (PCOS) is an important confounding factor in the assessment of ovarian reserve [20,21], we subdivided ovarian factors into PCOS and other ovarian disorders. Furthermore, genital tuberculosis (GTB) most commonly affects the bilateral fallopian tubes, accounting for 90–100%, followed by the endometrium and ovaries [22,23,24], so most of the patients should not be classified as GTB since they had the patency of at least one fallopian tube. Some women had two or more etiologies of infertility. The etiology of infertility was recorded and classified for each woman.

### 2.3. Ovulation Induction Protocols and Management in the IUI Program

In addition to natural cycles without ovarian stimulation, women may also undergo ovarian stimulation to induce ovulation, including clomiphene citrate, letrozole, gonadotrophin (human menopausal gonadotropin (HMG), urofollitropin (Zhuhai Livzon, Zhuhai, China) or recombinant follicle-stimulating hormone (rFSH, Gonal-F, Merck Serono S.p.A., Darmstadt, Germany)) from day 3 to 5 of the menstrual cycle for a variable duration depending on the follicle diameter. Follicles were monitored with the use of transvaginal sonography (TVS) by gynecologists in our department. In both natural and ovary-stimulated cycles, we monitored the follicle from day 10 to 12 of the menstrual cycle and then repeated every 2 or 3 days based on the follicle diameters. Women were advised to cancel a cycle if three or more dominant follicles were present. We performed ovulation triggering with the use of the intramuscular injection of 4000–10,000 IU urinary human chorionic gonadotrophin (hCG) (Zhuhai Livzon, Zhuhai, China) or 0.25 mg recombinant hCG (Merck Serono S.p.A., Darmstadt, Germany) when at least one dominant follicle diameter was assessed to be ≥18 mm. Insemination was performed 36–38 h after triggering.

### 2.4. Semen Preparation and Insemination

On the day of insemination in AIH cycles, semen was collected at the laboratory after 2–7 days of abstinence and prepared with density-gradient centrifugation after liquefaction. The volume of washed semen sample used for insemination was 0.5 mL. In AID cycles, the sperm was obtained directly from the sperm bank. After the evaluation of count and motility of spermatozoa, the insemination procedure was performed by a gynecologist.

### 2.5. Luteal Phase Support

Luteal phase support was applied in all women, commencing on the insemination day. It consisted of 200 mg/d progesterone soft capsules (Utrogestan; Besins, Brussels, Belgium) or 20 mg/d dydrogesterone (Duphaston; Abbott, Hoofddorp, The Netherlands) or 90 mg/d progesterone sustained-release vaginal gel (Merck Serono S.p.A., Darmstadt, Germany) for 14 days. A serum β-hCG assay was performed on the 14th day after insemination to assess whether pregnancy had occurred. If the result was positive, TVS was performed after 14–16 days, and luteal phase support would be maintained until 10 to 12 weeks of gestation.

### 2.6. Primary and Secondary Outcomes

The primary outcome of this study was ovarian reserve and live birth rate. Every woman had undergone TVS on the 2–4th day of the menstrual cycle for an antral follicle count (AFC). Follicles with a diameter of 2–9 mm in both ovaries were counted. A basal serum FSH and anti-mullerian hormone (AMH) were also performed to evaluate the ovarian reserve on the 2–4th day of the menstrual cycle. Live birth was defined as the birth of at least one live baby after 28 weeks of gestation [25]. Secondary outcomes included biochemical pregnancy rate, clinical pregnancy rate, miscarriage rate, multiple pregnancy rate, preterm birth rate, cesarean section rate, mean gestational age, mean birth weight and maternal and neonatal complications. Biochemical pregnancy was defined as a serum level of hCG of more than 10 mIU/mL. Clinical pregnancy was defined as the presence of an intrauterine gestational sac seen on TVS 4–6 weeks after ovulation, regardless of whether there is a heartbeat. Miscarriage was defined as the loss of pregnancy before 28 gestational weeks. Multiple pregnancy was defined as a pregnancy with more than one embryo or fetus [25]. Maternal and neonatal complications included mean gestational age, preterm birth, low birth weight, fetal macrosomia, gestational diabetes mellitus (GDM), pregnancy-induced hypertension (PIH), placenta previa, fetal growth restriction (FGR), premature rupture of membranes (PROM), intrahepatic cholestasis of pregnancy (ICP), pneumonia of newborn and birth defect.

### 2.7. Statistical Analysis

Statistical analysis was performed with SPSS 26.0 and R studio 3.5.3. Continuous variables were presented as the mean ± standard deviations (SD) for normal distribution and as the median (interquartile range, IQR) for skewed distribution data, and were compared via the Student’s *t*-test or Mann–Whitney U Test, respectively. Categorical variables were presented as relative frequencies and were compared with the Chi-square test or Fisher’s exact tests. The propensity score is the probability of treatment assignment conditional on observed baseline characteristics. The propensity score allows one to design and analyse an observational (non-randomized) study to mimic some of the characteristics of a randomized, controlled trial. The propensity score matching (PSM) can remove confounding effects in multiple clinical analyses, enabling balanced and unbiased comparison [26]. Unbalanced covariates were selected to estimate the propensity score by logistic regression, for their potential to be confounders for the ovarian reserve and pregnancy outcomes of IUI. Women of LTBI and non-LTBI groups were propensity-matched in a 1:2 ratio without replacement. A two-sided *p*-value < 0.05 was considered statistically significant.

### 2.8. Ethics Approval and Consent to Participate

This retrospective analysis was approved by the Ethics Committee of Tongji Hospital, Tongji Medical College, Huazhong University of Science and Technology (approval number:TJ-IRB20210856; approval date: 23 August 2021). The informed consent was waived by the Ethics Committee of Tongji Hospital, Tongji Medical College of Huazhong University of Science and Technology. All methods were carried out in accordance with relevant guidelines and regulations (declarations of Helsinki).

## 3. Results

**Figure 1:**
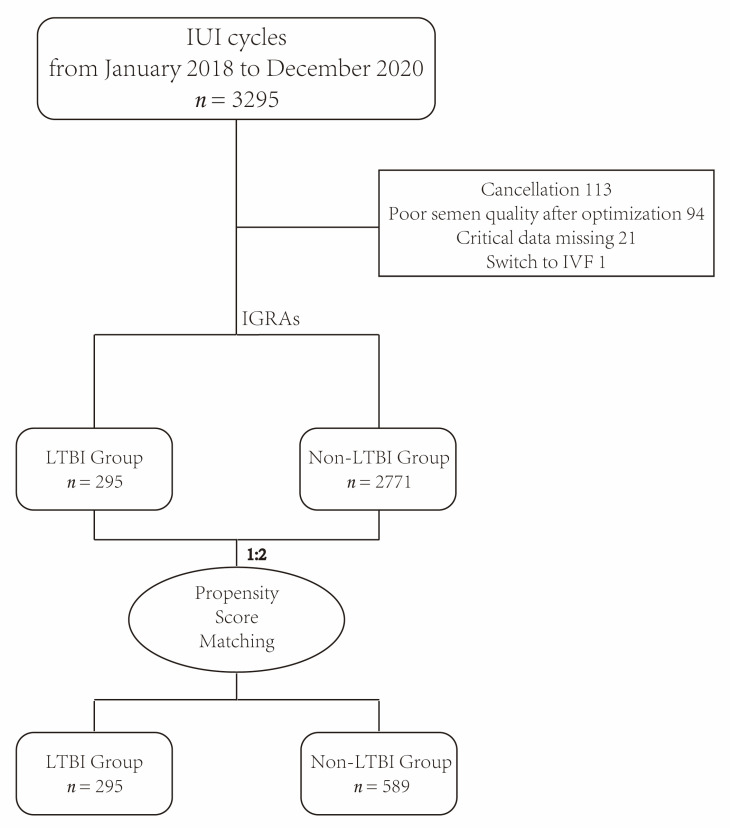
Flow chart of the selection of cases for inclusion in this study.

From January 2018 to December 2020, there were 3295 IUI cycles (2459 AIH cycles and 836 AID cycles) undergoing the IGRA examination in our center, and 3066 IUI cycles (2237 AIH cycles and 829 AID cycles) were finally included in this study (Figure 1).

Among the 3066 IUI cycles, 9.6% (295/3066) of women were diagnosed with LTBI. Table 1. shows the demographic characteristics of these women. Before PSM, women with LTBI were older (29.95 ± 3.37 mIU/mL vs. 29.47 ± 3.44 mIU/mL; *p*-value 0.021) than the non-LTBI group and had a higher rate of PCOS (35.6% vs. 27.9%, *p*-value 0.006), a lower rate of endometriosis (0.3% vs. 3.1%, *p*-value 0.007), a lower rate of chromosome abnormalities (7.1% vs. 11.2%, *p*-value 0.034) and a lower rate of primary infertility (79.3% vs. 84.8%, *p*-value 0.013). Subsequent PSM minimized the imbalance of these baseline characteristics.

**Figure 2:**
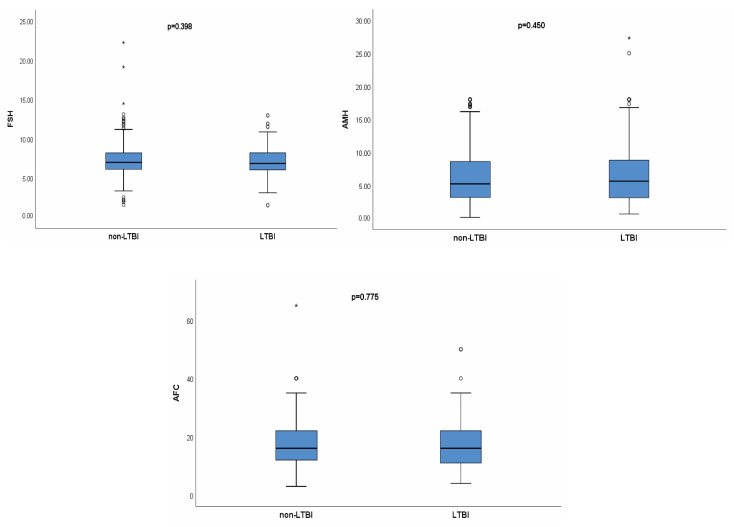
Comparisons of basal FSH, AMH and AFC between LTBI group and non-LTBI group. ◯: mild outliers (lower quartile minus 1.5IQR, or upper quartile plus 1.5IQR); *: extreme outliers (lower quartile minus 3IQR, or upper quartile plus 3IQR).

On the ovarian reserve, after PSM, there were no significant differences in the serum FSH (7.16 ± 1.67 mIU/mL vs. 7.27 ± 1.90 mIU/mL; *p*-value 0.398), AMH (median (IQR): 5.66 (3.15, 8.88) vs. 5.25 (3.20, 8.63); *p*-value 0.450) and AFC (median (IQR): 16.00 (11.00, 22.00) vs. 16.00 (11.50, 22.00); *p*-value 0.775) between the two groups (Figure 2). On the pregnancy outcomes, after PSM, the data showed that women who had LTBI had a trend toward a lower biochemical pregnancy rate (12.9% vs. 17.7%, *p*-value 0.068), lower clinical pregnancy rate (10.8% vs. 15.1%, *p*-value 0.082), lower live birth rate (8.1% vs. 12.1%, *p*-value 0.076) and higher miscarriage rate (25.0% vs. 20.2%, *p*-value 0.573) with no significant differences. There were no significant differences in other secondary outcomes between the two groups (Table 2). In addition, all women in the LTBI group did not develop active TB during pregnancy.

## 4. Discussion

With the rapid development of ART, more and more women with infertility have realized their desire to have offspring, and how to improve clinical outcomes of ART has been one of the most important topics worldwide recently. According to a previous study, about a quarter of the global population is estimated to have been infected with TB, most of whom are LTBI and at risk of developing active TB [27]. LTBI has neither TB-related symptoms nor evidence of active TB on bacteriology and imaging. In addition, an important feature of LTB is the loss of culturability. Therefore, the current diagnosis of LTBI relies on the detection of specific immune responses produced by the body to the TB antigen, so as to make the diagnosis of whether the body is infected with TB [3,28]. Anti-TB antibody tests and tuberculin skin tests (TST) were once widely used in clinical TB screening, but the accuracy of detection was affected by their low sensitivity and specificity. In recent years, IGRA, which measures cellular immune responses to the TB-specific proteins, early secreted antigenic target 6 and 10-kDa culture filtrate protein, has become a new method for in vitro immune detection of TB [29]. IGRA (the QuantiFERON-TB Gold In-Tube (QFT-GIT) assay and the T-SPOT.TB assay) is not affected by human CD4+T lymphocytes and has higher sensitivity and specificity. Additionally, it excludes the interference of Bacillus Calmette-Guerin (BCG) vaccine and most non-pathogenic mycobacteria [30,31,32,33]. The advantages of IGRA in TB diagnosis have been confirmed by many studies, especially for countries with a high burden of TB such as China, which are widely vaccinated with BCG. A recent prospective cohort study in America found that 7.7% (25/323) of infertile women were diagnosed with LTBI by IGRA, and the incidence of recurrent spontaneous abortion (RSA) and Asherman syndrome in LTBI women was significantly higher than non-LTBI women [34]. In our study, the prevalence of LTBI diagnosed by IGRA in infertile women was 9.6% (295/3066), which was higher than in America [1,34]. Furthermore, DNA PCR is also being used for diagnosing LTB in asymptomatic infertile women, particularly when multi-gene amplification is performed [12].

TB damage to tubal function and the endometrium in histologically confirmed GTB is well documented. Some evidence suggesting that GTB may also affect ovarian reserve adversely, with papers being published regarding the influence on AMH [13,14,35,36,37,38,39]. In addition, the success of implantation depends on a competent embryo, a receptive endometrium and an effective crosstalk between the two. The normal immune system function at the maternal–fetal interface plays an important role [40,41]. Previous studies have found that patients with LGTBI have fewer oocytes, poorer embryo quality and lower sub-endometrial blood flow [42] and that immune dysregulation can also adversely affect the endometrium and oocytes, which has been confirmed by a case-control study of concomitant autoimmunity in endometriosis [43]. Patients with LTBI also have long-term immune dysregulation, manifested by alterations of pro-inflammatory and anti-inflammatory cytokines, imbalanced immune cell populations and altered cytokine profiles [44,45]. We speculate that it may also affect important early reproductive steps such as oocyte maturation and embryo implantation, but there is still a lack of enough evidence to prove this conjecture. However, our study shows that infertile women with LTBI had no significant difference in ovarian reserve compared with the non-LTBI group after excluding the confounders such as PCOS, endometriosis and age [20,21,46,47], and LTBI did not raise the incidence of maternal and neonatal complications in infertile women undergoing IUI, which is not the same as previous studies [14,42]. We considered the main reason for these results is that positive IGRA indicates LTBI but does not indicate GTB. GTB has a low detection rate when the common diagnostic modalities of microbiological culture, histopathology and laparoscopy are used [13,34], and GTB most commonly affects the bilateral fallopian tubes, accounting for 90–100%, followed by the endometrium and ovaries [22,23,24]. Actually, every woman in our department had undergone HSG or HyCoSy examination before IUI, which confirmed the patency of at least one fallopian tube. Therefore, most LTBI women should not be classified as GTB. Another possible reason is that the sample size was relatively small, so our study only showed a trend in adverse pregnancy outcomes but with no significant differences.

Despite all this, the IGRA examination before ART can not only clarify whether there was TB infection in the past, providing a reference for ART clinical decision making, but also has important reference significance for the exclusive diagnosis of cough, fever and other symptoms during pregnancy. Changes in women’s immune systems during pregnancy would make them more susceptible to active TB, and delayed or missed diagnosis of TB in women undergoing active ART treatment can be potentially life threatening for the mother and devastating for conceived pregnancies [12]. Systemic hematogenous dissemination of TB after oocyte pick-up, transplacental fetal transmission of TB, and adverse maternal and neonatal outcomes such as preterm birth, miscarriage, and maternal and infant death have been reported [37,38,48,49,50,51,52,53,54]. Therefore, the Swedish national guidelines recommend that pregnant women, especially those in high-risk areas, should be screened for TB systematically, which is helpful for early detection and treatment of LTBI and asymptomatic active TB [55].

Since there is currently no effective method to precisely predict whether LTBI women will develop active TB during pregnancy, whether these women need preventive anti-TB therapy before ART is still controversial. A previous study has indicated that untreated participants with a positive IGRA result have a 10.8-fold higher rate of progression to active TB [56]. Some studies showed that anti-TB treatment can significantly improve the clinical outcomes of women undergoing long-term infertility or recurrent IVF failure with LGTBI [14,42]. However, some experts believe that if LTBI is found before or during pregnancy, it can be treated after childbirth, and pregnancy will not affect the course of TB [37]. Many studies certainly suggested that preventive treatment is recommended for people who are at high risk for the progression of LTBI to active TB such as migrants, HIV-positive people, those on immunosuppression therapy for tumors, necrosis factor α inhibitors, or glucocorticoids, those receiving organ or hematologic transplantation and those who have recently been exposed to infection [4,29,34,55].

To our knowledge, this is the first clinical study to compare the ovarian reserve and pregnancy outcomes of infertile women undergoing IUI with and without LTBI. However, there are still several limitations in our study. First, it was a retrospective cohort study conducted in a single center and limited by the relatively small sample size, which might introduce a selection bias. Additionally, laparoscopic or hysteroscopic surgery had not been performed for acid-fast bacilli staining, microbiological culture and histopathology examination in women with LTBI, so it was hard to accurately determine whether there was GTB. Therefore, more prospective multi-center studies with a larger number of women and a comparison among the LTBI and non-LTBI women undergoing IVF need to be conducted in the future.

## 5. Conclusions

In conclusion, it appears that there was no significant difference in ovarian reserve between women with and without LTBI. Women with LTBI tended to have worse pregnancy outcomes after undergoing IUI, but the difference was not significant. Further studies are needed to confirm these findings.

## Figures and Tables

**Table 1:** Demographic characteristics of the cohort.

	Before Propensity Score Matching			After Propensity Score Matching		
	Non-LTBI (*n* = 2771)	LTBI (*n* = 295)	*p*-Value	Non-LTBI (*n* = 589)	LTBI (*n* = 295)	*p*-Value
Age (years)	29.47 ± 3.44	29.95 ± 3.37	0.021 *	29.76 ± 3.47	29.95 ± 3.37	0.437
Etiology of Infertility						
PCOS (%)	27.9 (774/2771)	35.6 (105/295)	0.006 *	34.3 (202/589)	35.6 (105/295)	0.702
Other ovary disorder (%)	6.4 (176/2771)	5.4 (16/295)	0.532	5.8 (34/589)	5.4 (16/295)	0.832
Fallopian tube and pelvic factor (%)	16.1 (446/2771)	12.5 (37/295)	0.111	17.1 (101/589)	12.5 (37/295)	0.075
Endometriosis (%)	3.1 (86/2771)	0.3 (1/295)	0.007 *	0.3 (2/589)	0.3 (1/295)	>0.999
Male factor (%)	56.4 (1562/2771)	52.5 (155/295)	0.208	54.2 (319/589)	52.5 (155/295)	0.649
Uterine factor (%)	8.8 (244/2771)	6.8 (20/295)	0.238	7.5 (44/589)	6.8 (20/295)	0.709
Chromosome factor (%)	11.2 (309/2771)	7.1 (21/295)	0.034 *	7.8 (46/589)	7.1 (21/295)	0.714
Unexplained (%)	22.5 (624/2771)	24.7 (73/295)	0.386	21.2 (125/589)	24.7 (73/295)	0.236
Type of IUI			0.078			0.965
AIH (%)	72.5 (2009/2771)	77.3 (228/295)		77.4 (456/589)	77.3 (228/295)	
AID (%)	27.5 (762/2771)	22.7 (67/295)		22.6 (133/589)	22.7 (67/295)	
Type of Infertility			0.013 *			0.191
Primary infertility (%)	84.8 (2351/2771)	79.3 (234/295)		75.4 (444/589)	79.3 (234/295)	
Secondary infertility (%)	15.2 (420/2771)	20.7 (61/295)		24.6 (157/912)	20.7 (61/295)	
Duration of Infertility (years)	3.00 (2.00, 4.00)	2.00 (2.00, 4.00)	0.845	3.00 (2.00, 4.00)	2.00 (2.00, 4.00)	0.740
BMI (kg/m^2^)	21.71 ± 3.01	21.77 ± 2.67	0.691	21.99 ± 3.10	21.77 ± 2.67	0.278
Ovulation Protocol			0.295			0.648
Ovarian stimulation (%)	86.6 (2401/2771)	88.8 (262/295)		89.8 (529/589)	88.8 (262/295)	
Natural ovulation (%)	13.4 (370/2771)	11.2 (33/295)		10.2 (60/589)	11.2 (33/295)	
Endometrial Thickness on HCG day (mm)	9.30 ± 2.04	9.32 ± 2.13	0.898	9.22 ± 2.04	9.32 ± 2.13	0.524

* *p*-value < 0.05.

**Table 2:** Pregnancy outcomes of intrauterine insemination with LTBI and with non-LTBI.

	Before Propensity Score Matching			After Propensity Score Matching		
	Non-LTBI (*n* = 2771)	LTBI (*n* = 295)	*p*-Value	Non-LTBI (*n* = 589)	LTBI (*n* = 295)	*p*-Value
Biochemical pregnancy (%)	15.1 (419/2771)	12.9 (38/295)	0.305	17.7 (104/589)	12.9 (38/295)	0.068
Clinical pregnancy (%)	12.7 (352/2771)	10.8 (32/295)	0.360	15.1 (89/589)	10.8 (32/295)	0.082
Live birth (%)	10.6 (294/2771)	8.1 (24/295)	0.185	12.1 (71/589)	8.1 (24/295)	0.076
Ectopic pregnancy (%)	0.3 (8/2771)	0.3 (1/295)	0.598	0.3 (2/589)	0.3 (1/295)	>0.999
Miscarriage (%)	16.5 (58/352)	25.0 (8/32)	0.221	20.2 (18/89)	25.0 (8/32)	0.573
Multiple pregnancy (%)	4.8 (17/352)	3.1 (1/32)	>0.999	1.1 (1/89)	3.1 (1/32)	0.461
Gestational age (weeks)	38.58 ± 1.59	38.88 ± 1.26	0.373	38.77 ± 1.47	38.88 ± 1.26	0.765
Mean birth weight (g)	3240.37 ± 504.02	3301.04 ± 509.07	0.571	3341.55 ± 492.06	3301.04 ± 509.07	0.730
Caesarean section (%)	65.0 (191/294)	70.8 (17/24)	0.561	71.8 (51/71)	70.8 (17/24)	0.925
Maternal and neonatal complications						
Preterm Birth (%)	5.1 (18/352)	3.1 (1/32)	0.943	4.5 (4/89)	3.1 (1/32)	>0.999
Gestational Diabetes Mellitus (%)	4.3 (15/352)	3.1 (1/32)	>0.999	4.5 (4/89)	3.1 (1/32)	>0.999
Pregnancy-induced Hypertension (%)	2.6 (9/352)	3.1 (1/32)	0.586	2.2 (2/89)	3.1 (1/32)	>0.999
Low Birth Weight (%)	5.4 (19/352)	6.3 (2/32)	>0.999	2.2 (2/89)	6.3 (2/32)	0.610
Fetal Macrosomia (%)	2.3 (8/352)	3.1 (1/32)	0.547	2.2 (2/89)	3.1 (1/32)	>0.999
Placenta Previa (%)	1.1 (4/352)	0	>0.999	1.1 (1/89)	0	>0.999
Premature Rupture of Membranes (%)	1.1 (4/352)	0	>0.999	1.1 (1/89)	0	>0.999
Intrahepatic Cholestasis of Pregnancy (%)	0.3 (1/352)	0	>0.999	1.1 (1/89)	0	>0.999
Fetal Growth Restriction (%)	0.3 (1/352)	0	>0.999	0	0	N/A
Birth Defect (%)	0.3 (1/352)	0	>0.999	0	0	N/A
Asphyxia of Newborn (%)	0.3 (1/352)	0	>0.999	1.1 (1/89)	0	>0.999
Pneumonia of Newborn (%)	0.3 (1/352)	0	>0.999	0	0	N/A

N/A, not applicable.

## Data Availability

The datasets generated and analysed during the current study are not publicly available due to individual privacy but are available from the corresponding author upon reasonable request.
